# Evaluating the Immunogenicity, Efficacy, and Effectiveness of Recombinant Zoster Vaccine for Global Public Health Policy

**DOI:** 10.3390/vaccines13030250

**Published:** 2025-02-27

**Authors:** Lucy R. Williams, Joachim Hombach, Melanie Marti

**Affiliations:** 1 MRC Centre for Global Infectious Disease Analysis, School of Public Health, Imperial College London, London W12 0BZ, UK; 2 Immunisation, Vaccines and Biologicals Department, World Health Organisation, Avenue Appia 20, 1211 Geneva, Switzerland

**Keywords:** vaccine, immunization, herpes zoster, varicella zoster virus, health policy

## Abstract

**Background**: Herpes zoster (HZ) is a painful neurocutaneous disease caused by the varicella-zoster virus. The recombinant zoster vaccine (RZV) is becoming increasingly incorporated into national vaccination schedules. We aimed to evaluate RZV from a global public health policy perspective. **Methods**: We performed a rapid review of studies evaluating the immunogenicity, efficacy, and effectiveness of RZV for protection against HZ and associated complications. We searched PubMed for English-language studies published between 7 August 2012 and 30 September 2023. Included studies reported vaccine efficacy or effectiveness against HZ and HZ-associated complications. Immunogenicity studies were included if they contributed to the understanding of RZV protection over time and/or co-administration with other vaccines. HZ outcomes were stratified by socio-demographic and clinical variables. **Results**: From 405 identified publications, 33 were eligible for the study. Most studies were conducted in the US (N = 12), across North America (N = 10), and Europe (N = 5), or across multiple locations across North America, Latin America, and Asia–Australia (N = 6). Vaccine efficacy against HZ in immunocompetent populations ranged between 90% and 97%, while effectiveness ranged between 71% and 86%. Protection stayed above 70% for at least 10 years, with no significant differences by age or ethnicity. **Conclusions**: RZV is effective in reducing the risk of HZ and its associated complications. Protection is long-lasting and the vaccine is suitable for older and immunocompromised populations. However, the decision to incorporate the vaccine into national policies depends on additional factors (e.g., cost-effectiveness), which may be difficult to characterize without an understanding of the global disease burden.

## 1. Introduction

Herpes zoster (HZ), or shingles, is a painful neurocutaneous disease caused by reactivation of the varicella-zoster virus (VZV) in the dorsal root ganglia [1,2]. It is characterized by a blister-like rash on one side of the body, which is commonly accompanied by radicular pain lasting 2–4 weeks. The pain associated with HZ can be debilitating and lead to depression, social withdrawal, and reduced quality of life, and places a large burden on patients, carers, healthcare systems, and employers [3,4].

A large proportion of the burden of HZ on healthcare systems is from HZ-associated complications, of which post-herpetic neuralgia (PHN) is the most common. PHN is a chronic neuropathic pain that persists after resolution of the acute phase of vesicular rash, which occurs in approximately one in five HZ patients [5]. If VZV reactivates in certain cranial nerves, it can also cause herpes zoster ophthalmicus (HZO) and this may lead to conjunctivitis, keratitis, iritis, uveitis, and vision loss [6]. Other complications include disseminated HZ (rash in ≥3 dermatomes), and neurological, visceral, or vascular diseases, including stroke and myocardial infarction (MI) [7].

Between 20% and 30% of the population are expected to develop HZ in their lifetime [8,9]. An estimated 14.9 million cases of HZ occurred globally in 2020 in individuals aged over 50 years, and this is predicted to increase to up to 19.1 million cases by 2030 [10]. The risk and severity of HZ increases with age, particularly after the age of 50 years, due to age-related decline in immunity [11]. Patients with immunosuppression and other severe comorbidities are also at higher risk of developing HZ than immunocompetent individuals of any age, and they are more likely to suffer from severe disease or HZ-associated complications [12,13]. Data availability on the incidence of HZ and its associated complications varies widely, with most studies conducted in North America and Europe. In a systematic literature review of HZ incidence worldwide in the population over 50 years, van Oorschot et al. found that the incidence of HZ ranged between 5.2 and 10.9 per 1000 person-years across North America, Europe, and the Asia-Pacific region [14], but, consistent with other studies, they found little to no data for the regions of Eastern Europe, the Middle East, South America, or Africa [15,16,17].

There are over a dozen candidate vaccines in clinical development for the prevention of HZ, and, to date, two vaccines have been licenced: Zoster Vaccine Live (ZVL, Zostavax^®^, Merck Sharp & Dohme B.V, Haarlem, The Netherlands) and the Recombinant Zoster Vaccine (RZV, Shingrix^®^, GlaxoSmithKline, Brentford, UK). ZVL demonstrated moderate vaccine efficacy of 51.3% during follow-up lasting a median time of 3.1 years [18,19]. Vaccine effectiveness in real-world settings, particularly over extended follow-up, was low, with one study reporting an effectiveness of 31.8% after 8 years post-vaccination [20]. Additionally, as it contains a weakened form of the VZV, it is contraindicated in immunocompromised individuals [20]. RZV is a recombinant (non-live) vaccine which contains the adjuvant AS01_B_ and recombinant VZV glycoprotein E (gE). It was licenced under a two-dose regimen by the U. S. Food and Drug Administration (FDA) in 2017 and the European Medicines Agency (EMA) in 2018 for adults 50 years or older and immunocompromised adults over the age of 18 years. ZVL has now been discontinued by the manufacturer, so many countries have already begun, or are currently in the process of transitioning to the use of RZV for their HZ vaccination programmes [3].

This rapid review aims to provide an up-to-date assessment of the evidence on the immunogenicity, efficacy, and effectiveness of RZV and how this differs across key clinical and socio-demographic characteristics. It may help to inform policymakers who are tasked with issuing guidance on RZV use on the benefits and potential risks in different population groups.

## 2. Materials and Methods

### 2.1. Search Strategy, Inclusion and Exclusion Criteria

We conducted our literature search in PubMed to identify randomized controlled trials (RCTs) and non-randomized studies evaluating the immunogenicity, efficacy, and effectiveness of RZV from 7 August 2012 to 30 September 2023. This date range was chosen because the first phase 2 trial results were published in August 2012 [21]. One reviewer (LRW) performed the study selection and data extraction. Included studies reported on protection against HZ, PHN, and other HZ-associated complications. Immunogenicity studies were included if they contributed to the understanding of continued vaccine protection over time and/or the breadth of immune response when RZV was co-administered with other vaccines. We allowed for any population characteristics and did not impose an age limit. We compare two doses of RZV to no RZV administration, and we only included publications written in English. Inclusion and exclusion criteria are provided in Table 1 and search terms are provided in Table 2. A systematic review of safety outcomes was beyond the scope of this report; however, we summarized the findings (reactogenicity, adverse events, and serious adverse events) of other reviews on the topic.

### 2.2. Data Extraction

Data extracted included the study methodology (design, sample size, location, duration, population characteristics) and study outcomes. Outcomes of interest were the incidence rate, relative risk (RR), and vaccine efficacy/effectiveness against HZ, PHN, hospitalization, and other HZ-associated complications (e.g., HZO, stroke, myocardial infarction). We stratified HZ outcomes by age, geographic ancestry/ethnicity (Asian, Black, Hispanic, White), sex, receipt of previous ZVL, immune status, co-administration with another vaccine, and time since vaccination. Immunogenicity outcomes included humoral and cellular immune responses.

Vaccine efficacy and effectiveness are presented as reported. If only RR was reported, if it had been calculated as the incidence rate ratio or hazard ratio, we calculated efficacy/effectiveness as 1-RR. Odds ratios were not converted to efficacy/effectiveness [22]. Where there were no cases in the vaccinated group, efficacy/effectiveness is given as 100% with no confidence intervals.

### 2.3. Bias Assessment

One reviewer (MM) independently assessed the quality, level of evidence, and risk of bias of all included studies (Supplementary Methods). The Cochrane Risk of Bias tool (RoB2) was used for RCTs and the Risk of Bias in Non-randomized Studies of Interventions (ROBINS-I) was used for observational studies. All studies, regardless of their methodological quality, underwent data extraction and estimates are presented.

## 3. Results

### 3.1. Summary of RZV Study Characteristics

**Figure 1:**
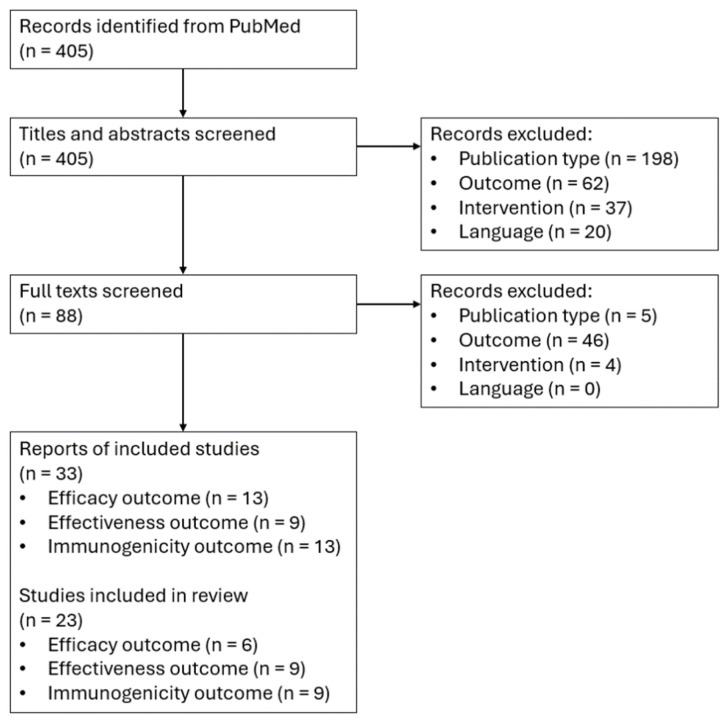
Study flow diagram.

A total of 405 publications were identified from the database search. After titles and abstracts were assessed for eligibility, 88 publications were appraised for eligibility based on the pre-specified inclusion and exclusion criteria. We identified 33 eligible publications from 23 studies evaluating the efficacy [23,24,25,26,27,28,29,30,31,32,33,34,35] (Table 3), effectiveness [36,37,38,39,40,41,42,43,44] (Table 4), and immunogenicity [30,31,45,46,47,48,49,50,51,52,53,54,55] (Table 5) of RZV (Figure 1). Most studies were conducted exclusively in the US (N = 12), across multiple countries in North America and Europe (N = 5), or across multiple locations across North America, Latin America, and Asia–Australia (N = 6). Six studies were randomized controlled trials, eight were open-label randomized trials, eight were cohort studies, and one was case–control. The overall quality of the included RCTs was high and most had a low risk of bias (Supplementary Table S1). While well executed, most of the observational studies had a moderate risk of bias (Supplementary Table S2).

### 3.2. Immunocompetent Populations

**Figure 2:**
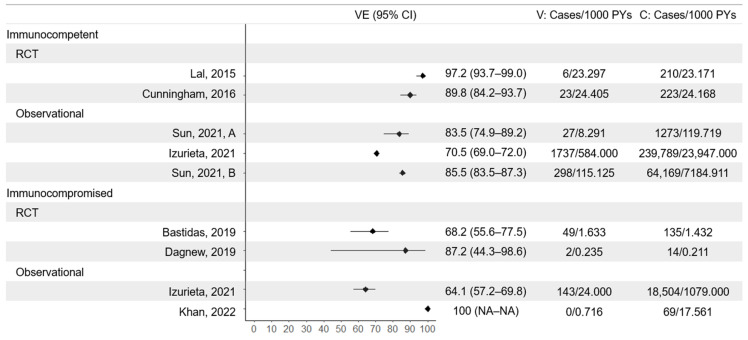
Vaccine efficacy and effectiveness against herpes zoster in immunocompetent and immunocompromised populations. Studies: Lal et al., 2015 (ZOE-50) [23], Cunningham et al., 2016 (ZOE-70 only) [24], Sun et al., 2021 (A) [36], Izurieta et al., 2021 (general immunocompromised status) [38], Sun et al., 2021 (B) [39], Bastidas et al., 2019 (hematopoietic stem cell recipients) [33], Dagnew et al., 2019 (Haematological malignancy patients) [35], Khan et al., 2022 (inflammatory bowel disease patients, 50–60 years age group, vaccine effectiveness calculated from hazard ratio) [42]. V = vaccine arm; C = control arm/unvaccinated; PYs = person-years.

Vaccine efficacy against HZ was evaluated in two pivotal phase III efficacy trials in immunocompetent populations over 50 years (ZOE-50 [23]) and 70 years (ZOE-70 [24]) (Figure 2). ZOE-50 demonstrated high efficacy against HZ (97.2%, 95% CI 93.7–99.0) after a mean follow-up of 3.5 years, while ZOE-70 demonstrated that protection is maintained in older populations (efficacy against HZ 89.8%, 95% CI 84.2–93.7, mean follow-up 3.7 years). Effectiveness in the real world is typically lower than efficacy in controlled clinical trials, and although this holds for RZV, the protection against HZ has been shown to be substantial. In immunocompetent populations, effectiveness against HZ has ranged between 70.5% (95% CI 69.0–72.0) [38] and 85.5% (95% CI 83.5–87.3) [39] in cohort studies conducted in the US.

### 3.3. Immunocompromised Populations

**Figure 3:**
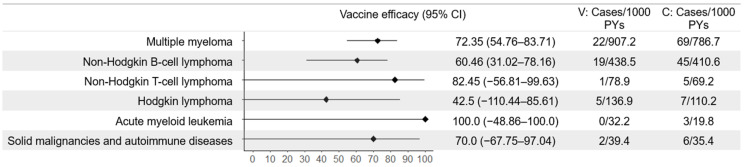
Vaccine efficacy against herpes zoster by disease in an immunocompromised trial population, reported by Stadtmauer et al. [34]. V = vaccine arm; C = control arm/unvaccinated; PYs = person-years.

Individuals with an immunosuppressive disease or on immunosuppressive therapy were excluded from the ZOE-50 and ZOE-70 efficacy trials. However, included participants with pre-existing potential immune-mediated diseases (majority psoriasis, rheumatoid arthritis, and spondyloarthropathy) were evaluated in a post hoc analysis [28]. In this population, vaccine efficacy against HZ was 90.5% (95% CI 73.5 to 97.5). Efficacy against HZ in truly immunocompromised populations was evaluated in two RCTs: ZOE-HSCT [33] and ZOE-039 [35]. The ZOE-HSCT study of hematopoietic stem cell transplant recipients found efficacy against HZ to be lower than in the comparative immunocompetent ZOE-50 and ZOE-70 populations. However, the protection was still moderate, with an estimated efficacy of 68.2% (95% CI 55.6–77.5) (Figure 2). In a post hoc analysis stratified by disease, the sample size was moderate for patients with multiple myeloma (*n* = 472) and non-Hodgkin B-cell lymphoma (*n* = 237), for which efficacy was estimated at 72.35% (95% CI 54.76–83.71) and 60.46% (95% CI 31.02–78.16), respectively [34]. For all other diseases (non-Hodgkin T-cell lymphoma, Hodgkin lymphoma, acute myeloid leukemia, and solid malignancies and autoimmune diseases), efficacy estimates ranged from 42.5% to 100% with wide confidence intervals (Figure 3). Zoster-039 found greater protection in a population of immunocompromised patients with hematological malignancies but, due to a small number of cases in both trial arms, with low precision (vaccine efficacy = 87.2%, 95% CI 44.3–98.6).

Vaccine effectiveness in immunosuppressed populations has been assessed in two studies of inflammatory bowel disease (IBD) patients [37,42], and one prospective cohort study of Medicare C beneficiaries [38]. In the Medicare C beneficiaries cohort, vaccine effectiveness was statistically significantly lower in the immunocompromised population (64.1%, 95% CI, 57.2–69.8), compared to the immunocompetent population (70.5%, 95% CI 69.0–72.0). Both studies of IBD patients found a reduction in the risk of HZ. Khan et al. [42] used their 50–60-year age group as a proxy for a population unlikely to have had prior HZ vaccine exposure. In this group, incidence in the vaccinated reduced from 3.93 to 0.00 per 1000 person-years, while in the >60-year age group (proxy for possibility of prior HZ vaccine exposure), incidence reduced from 4.57 to 1.80 per 1000 person-years. This corresponds to 100% (95% CI not calculable) and 61% (95% CI 20–81) effectiveness against HZ in the younger and older age groups, respectively. Among a similar population of IBD patients, Kochhar et al. showed a reduction in the odds of HZ by 64% (95% CI 44–77) [37]. While multiple studies have shown that protection against HZ is lower in immunocompromised populations than immunocompetent ones, protection is still substantial.

### 3.4. Protection Against HZ Complications and Hospitalization

**Figure 4:**
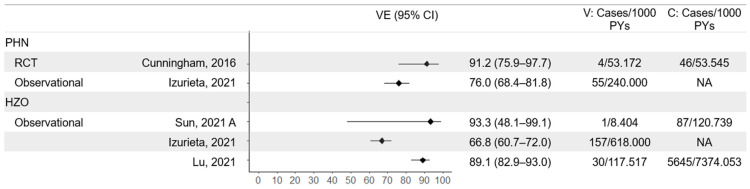
Vaccine efficacy and effectiveness of RZV against post-herpetic neuralgia (PHN) and herpes zoster ophthalmicus (HZO). Studies: Cunningham, 2016 [24], Izurieta, 2021 [38], Sun, 2021 (A) [36], Lu, 2021 [40]. VE = vaccine efficacy/effectiveness; V = vaccine arm; C = control arm/unvaccinated; PYs = person-years.

Pooled analysis of the ZOE-50 and ZOE-70 studies gave a larger sample size in which to investigate protection against PHN and other complications. In the >50-year-old vaccination cohort, PHN occurred at a rate of 0.1 cases per 1000 person-years in the vaccine arm and 0.9 cases per 1000 person-years in the placebo arm, which translated to an efficacy of 91.2% (95% CI 75.9 to 97.7) [24] (Figure 4). Only one non-PHN complication, ophthalmic disease, was recorded in the vaccine arm compared to sixteen in the placebo arm, in over 50,000 person-years of follow-up in each arm [25]. This gave an efficacy of 93.7% (95% CI 59.5 to 99.9) against non-PHN complications. Hospitalizations were also rare, with no cases observed in the RZV arm over 24,440 person-years and 5 in 24,631 years follow-up in the placebo arm. This gave a high estimated vaccine efficacy against hospitalization with wide confidence intervals (100%, 95% CI -9.9–100.0), which is consistent with ZOE-HSCT, in which the hazard ratio of hospitalization was 0.15 (95% CI, 0.03–0.68) [33]. Protection against PHN and HZO have also been shown to be high in real-world settings, with estimates ranging between 66.8% [38] and 93.3% [36] for HZO and one study reporting vaccine effectiveness of 76.0% for PHN. Again, these estimates are similar to the two-dose vaccine effectiveness against HZ (70.1%, 95% CI 68.6–71.5), suggesting negligible additional protection against complications after development of HZ.

The risk of myocardial complications is also lower in HZ patients who have previously been vaccinated with RZV. In one retrospective case–control study, the elevated risk of stroke in HZ patients compared to non-HZ patients (OR 1.93, 95% CI 1.57–2.40) was reduced by 43% in those who had previously been vaccinated with RZV (OR vaccinated HZ patient vs. unvaccinated HZ patient 0.57, 95% CI 0.46–0.72) [44]. Increased risk of myocardial infarction in HZ patients was also reduced by 28% in those vaccinated with RZV-vaccinated HZ (OR 0.82 95% CI, 0.74–0.92) [43].

### 3.5. Differences in Protection Against HZ Across Populations

#### 3.5.1. Age

**Figure 5:**
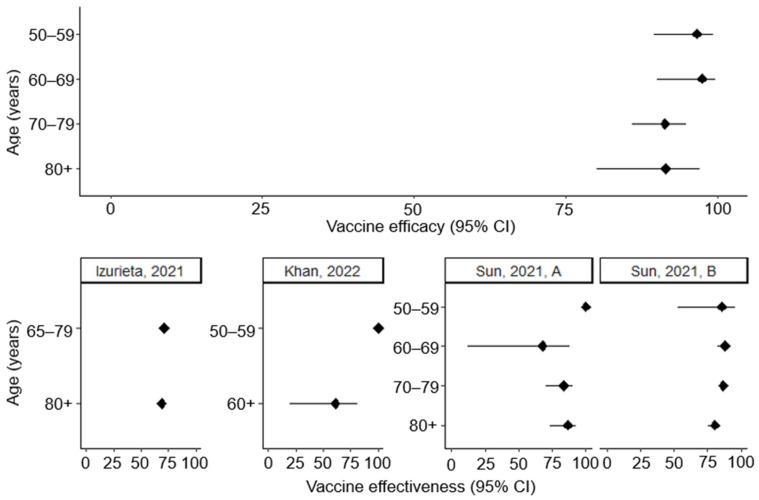
Vaccine efficacy and effectiveness against herpes zoster by age. Vaccine efficacy estimates taken from ZOE-50 study [23] for age groups 50–59 years and 60–69 years, and ZOE-50/70 for age groups 70–79 years and 80+ years [24]. Khan, 2022 vaccine effectiveness calculated from hazard ratio [42]. Other studies: Sun, 2021 (A) [36], Izurieta, 2021 [38], Sun, 2021 (B) [39].

In pooled ZOE-50/70 analysis, there was a slight decrease in efficacy with age, but it was statistically insignificant and estimates kept above 90% in all age groups (vaccine efficacy 50–59 years = 96.6% (95% CI 93.7–99.0), vaccine efficacy ≥80 years = 91.4 (95% CI 80.2–97.0) [24] (Figure 5). In observational studies, there has been mixed evidence for a slight reduction in people over 80 years. A retrospective cohort study conducted in the US by Sun et al. [39] found effectiveness in the ≥80-year-old population to be slightly lower (vaccine effectiveness = 80.3%, 95% CI 75.1–84.3) than in the population aged 50 to 79 years (vaccine effectiveness = 85.5%, 95% CI 83.5–87.3). Despite this, other observational studies have found no statistically significant difference across age groups [36,38].

#### 3.5.2. Ethnicity

**Figure 6:**
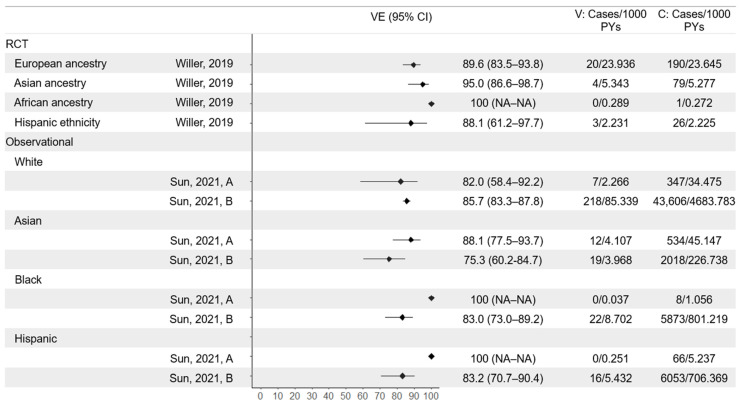
Vaccine efficacy and effectiveness of RZV against herpes zoster by geographic ancestry/ethnicity. RCT results reported in Willer et al. [26], observational results from Sun, 2021 (A) [36], and Sun, 2021 (B) [39]. Confidence intervals were not reported for subgroups with no cases in vaccinated cohort. VE = vaccine efficacy/effectiveness; V = vaccine arm; C = control arm/unvaccinated; PYs = person-years.

Post hoc analyses of ZOE-50/70 participants stratifying by geographic region and ethnicity found no statistically significant differences across either metric, although the sample size and therefore precision of estimates was low for some subgroups (Figure 6) [26,29]. Similarly, subgroup analyses of observational studies identified no statistically significant differences by ethnicity, but in some subgroups, there were no cases in the vaccinated cohort so confidence intervals could not be determined [36,39].

#### 3.5.3. Sex

Post hoc analyses of data from the ZOE-50/70 trials demonstrated no impact of sex on efficacy of RZV [26,29]. Efficacy against HZ was >90% in both female and male participants in the ZOE-50/70 populations. In the pooled ZOE-50/70 analysis, efficacy against PHN was 91.5% (95% CI: 65.7–99.1) and 83.3% (95%CI 24.8–98.2) in female and male participants, respectively [26]. Sun et al. [39] reported similar estimates of vaccine effectiveness by sex (adjuvanted vaccine effectiveness in females = 84.7% (95% CI 82.2–87.0) and males = 86.8 (95% CI 83.3–89.6)).

### 3.6. Duration of Protection

**Figure 7:**
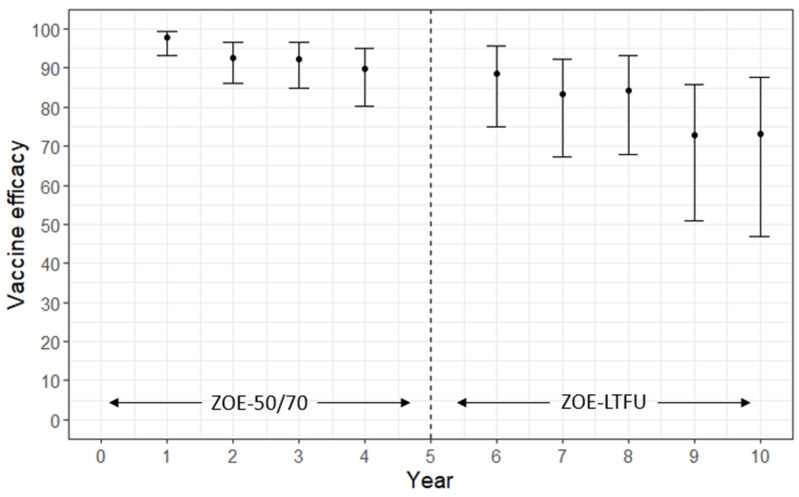
Vaccine efficacy (VE) against herpes zoster in the ZOE-50/70 and ZOE-LTFU studies over time since 1 month post-second dose reported in Strezova et al. [31]. No vaccine efficacy estimate given for year 5 due to switch between studies. In ZOE-50/70, VE was estimated using placebo control as comparator. In ZOE-LTFU, VE was estimated using historic controls from ZOE-50/70 placebo arm.

In the pooled ZOE-50/70 studies, vaccine efficacy stayed above 89% across the four-year follow-up period [31]. The ZOE-LTFU study is a long-term follow-up study of a subset of the ZOE-50/70 populations that compares the incidence of HZ in the ZOE-50/70 vaccine arm to historical controls from the placebo arm. In the most recent ZOE-LTFU interim analysis, eligible participants had been followed up for a mean of 5.6 years to 9.6 years post-vaccination [31]. The vaccine efficacy against HZ over this time period was 81.6% (95% CI 75.2–86.6). Annual estimates over time showed a gradual decline, from 88.5% (95% CI 74.9–95.6) at Y6 to 84.2% (95% CI 67.9–93.1) at Y8 and 73.2% (95% CI 46.9–87.6) at Y10 (Figure 7). Humoral immune responses were over 5-fold greater than pre-vaccination levels through 10 years post-vaccination. They were relatively constant up to Y8 but decreased slightly into Y9. Cell-mediated immune responses remained stable and over 6-fold above the pre-vaccination level through Y10. This is consistent with other long-term immunogenicity studies which have found humoral and cell-mediated immune responses to stay above pre-vaccination levels for up to 10 years after vaccination [53,55]. No vaccine effectiveness studies that evaluated the change in effectiveness over time were identified, but the study with the longest follow-up (median 3.1 years) found a vaccine effectiveness of 70.1% (95% CI 68.6–71.5) [38].

### 3.7. Prior Vaccination for HZ

**Figure 8:**
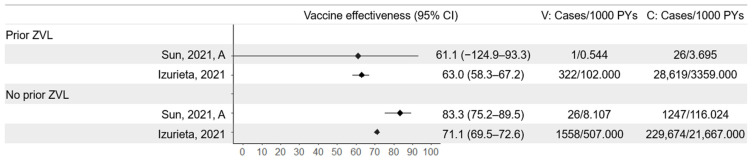
Vaccine effectiveness against herpes zoster in populations who had and had not previously been vaccinated with Zoster Vaccine Live (ZVL, Zostavax). Studies: Sun, 2021 (A) [36], Izurieta, 2021 [38]. V = vaccine arm; C = control arm/unvaccinated; PYs = person-years.

To date, no studies have evaluated efficacy against HZ in participants who have previously experienced HZ or who have been vaccinated with ZVL. Izurieta et al. compared the effectiveness against HZ in Medicare beneficiaries who had and had not received ZVL vaccination within the 5 years leading to study initiation [38]. Beneficiaries unvaccinated with RZV who had previously received ZVL had a lower incidence of HZ than those who had not previously received ZVL (prior ZVL = 8.52 cases per 1000 person-years, no prior ZVL = 10.60 cases per 1000 person-years) (Figure 8). RZV provided a protective effect against HZ regardless of prior ZVL status but had greater impact on those not previously vaccinated with ZVL (effectiveness of RZV in participants with prior ZVL = 63.0%, 95% CI 58.3–67.2), effectiveness of RZV in participants without prior ZVL = 71.1%, 95% CI 69.5–72.6). Sun et al. evaluated RZV effectiveness in study participants with and without vaccination with ZVL in the year before study initiation [36]. The sample size for the population vaccinated with RZV who had received ZVL was small and only one case occurred within this group. This led to wide confidence intervals for the effectiveness estimate, making conclusions from this study on the relative protection of RZV with and without prior ZVL difficult to draw.

### 3.8. Co-Administration

RZV is safe and effective when co-administered at different anatomic sites with other adult vaccines. In a cohort study in Southern California [41], RZV was administered to 28,353 and 12,898 individuals with and without co-administration, and the risk of HZ was not significantly different between the groups (HR HZ co-administration vs. no co-administration = 0.75 (95% CI, 0.53–1.08)). The majority of concomitant vaccine recipients only received one other vaccine with RZV (86.6%), with the most common being influenza vaccines (65.9%), pneumococcal vaccines (20.2%), and Td/Tdap (tetanus, diphtheria/tetanus, diphtheria, and pertussis) vaccines (12.3%). This is the only study to date that has evaluated vaccine effectiveness of RZV with concomitant vaccination.

However, the immunogenicity of the RZV and the following vaccines are unaffected by co-administration: 13-valent pneumococcal conjugate vaccine (PCV13) [48], 23-valent pneumococcal polysaccharide vaccine (PPSV23) [46], quadrivalent seasonal inactivated influenza vaccine (IIV4) [45], COVID-19 mRNA-1273 [49], and reduced-antigen-content diphtheria-tetanus-acellular pertussis vaccine (Tdap) (excluding one of the 5 Tdap antigens which did not meet the humoral response non-inferiority criterion) [47] (Table 5). Sequential administration of different vaccines containing the same AS01-adjuvant system was hypothesized to have the potential to cause immune interference [50], but, in a phase 2 study reporting on the immunogenicity of a candidate AS01-adjuvanted vaccine containing four surface proteins from non-typable Hemophilus influenzae and Moraxella catarrhalis (NTHi-Mcat), no evidence was found [50].

## 4. Discussion

This review found that RZV is both efficacious and effective for the prevention of HZ and its complications. Efficacy in immunocompetent populations was 97% in those over 50 years and 90% in those over 70 years. While protection is lower in immunocompromised populations, it is still substantial, with efficacy estimates ranging between 68% and 87%. There is no clear evidence of a reduction in protection with age or for differences across geographies, sexes, or ethnicities, but more data on subgroups would be valuable to yield more precise estimates. The vaccine provides protection when ZVL had previously been administered and vaccine efficacy remains over 70% through at least 10 years of follow-up.

Our findings are consistent with other reviews of RZV [56,57,58,59,60,61,62]. Xia et al. performed a systematic review and meta-analysis to directly compare both the RZV and ZVL vaccines [56]. They found RZV to be highly efficacious in a population over 60 years, and superior to ZVL across age and genders (relative vaccine efficacy: 84%, 95% CI: 53–95, and relative vaccine effectiveness: 49%, 95% CI: 21–67). Mbinta et al. found a conservative real-world effectiveness of ZVL against HZ of 46%, 95% CI: 42–49) and a higher post hoc estimate for RZV effectiveness of 79·2% (95% CI: 57·6–89·7) [58]. However, at the time of writing, few studies had evaluated the effectiveness of RZV for protection against HZ and its complications. In a more recent systematic review and meta-analysis, Zeevaert et al. illustrated high efficacy and effectiveness of RZV for HZ in immunocompetent and immunocompromised populations (VE immunocompetent 94%, 95% CI: 79–98, VE immunocompromised 70%, 95% CI: 53–81) [61]. Yet, they note that even with high levels of protection, the number needed to vaccinate in immunocompetent populations is high for HZ, and higher still for PHN. This is an important consideration in areas where the incidence of HZ is low or unknown, such as Eastern Europe, the Middle East, South America, and Africa [14]. In these areas, more targeted vaccination campaigns for immunocompromised and older populations at greater risk may be favourable, also from a perspective of cost-effectiveness.

Our review adds to these studies by providing the most up to date and comprehensive evaluation of the efficacy, effectiveness, and immunogenicity of RZV. No reviews have included an analysis of vaccine efficacy or immunogenicity for longer than 7 years post-vaccination [61], and we provide in-depth looks at key considerations for policymakers, such as the differences in protection across age groups, ethnicities, and immunocompromising conditions.

As the incidence and risk of complications for HZ increases with age, long-term protection is an important feature of a vaccine. Vaccine efficacy and humoral and cell-mediated immune responses gradually declined over time, but the evidence presented in this review supports protection for at least 10 years after vaccination. Furthermore, the decline in immune responses is slow and modelling of immunogenicity data suggests that both cellular and humoral immune responses may persist as long as 20 years after initial vaccination [53]. To add, a recently published abstract (See Abstracts at CIDSCON 2024. Journal of Clinical Infectious Diseases Society. 2(3):163–292, Jul-Sep 2024. ABSTRACT 100: Sammya Bhowmick et al. 2024. Adjuvanted Recombinant Zoster Vaccine—The First Vaccine to Provide Durable Protection against HZ in All Age Ranges ≥50 Years: Final Efficacy and Safety Analysis after 11 Years of Follow-up) suggests annual vaccine efficacy against HZ at Y11 being 82.0% (95% CI 63.0–92.2) in participants ≥50 years and 72.0% (95% CI 33.4–89.8) in those ≥70 years. While this suggests a booster dose may not be necessary for many individuals, a single booster dose does elicit strong humoral and cell-mediated immune responses [53]. This may be beneficial for subpopulations that experience a higher rate of immune waning. In the ZOE-50/70 trials, there was a faster decay of gE-specific cell-mediated immunity with older age in the first four years after vaccination [24], and in a study comparing the longevity of immune responses in RZV and ZVL recipients over 5 years post-vaccination, older age had a negative effect on the persistence of anti-gE antibody levels and avidity after 2 years post-vaccination [55]. RZV-induced protection against HZ is long lasting but continued research into the duration of protection and differences across populations will be useful for informing vaccination policies.

We did not identify any publications reporting on the efficacy or effectiveness of RZV in people who had previously experienced an HZ episode. However, the immunogenicity of RZV in patients ≥50 years after prior HZ disease was studied in an observational study in Canada and Russia [63]. Over 90% of participants reached the primary outcome of a four-fold increase in anti-gE antibodies one month after the second vaccination. Considering this immunogenicity evidence and the additional protection provided by RZV for patients who had previously received ZVL, vaccination regardless of prior infection status is likely to be beneficial.

Strengths of this review include the broad scope, including both RCTs and observational studies without limitation on the populations included. We went into depth on stratification by immune status, age, ethnicity, sex, and prior receipt of ZVL and considered additional outcomes such as hospitalization, PHN, HZO, and myocardial complications. These are important features for policymakers to consider when making vaccine recommendations. Limitations of our review include the small sample size for some stratifications of protection against HZ. For example, there were no vaccinated cases in people of Black or Hispanic ethnicity in observational studies evaluating vaccine effectiveness and few vaccinated cases in people of non-European ancestry in clinical trials evaluating vaccine efficacy. This makes differences in protection across ethnicities difficult to assess, and therefore an area in which further research would be valuable. Furthermore, estimates for immunocompromised populations are for a small subset of conditions, and we did not stratify by other specific comorbidities, such as HIV or rheumatoid arthritis. Finally, as we performed a rapid review, it does not comply fully with the PRISMA guidelines.

A systematic review of the reactogenicity and safety of RZV was beyond the scope of this review, but other reviews have found RZV to be safe in both RCTs and observational studies [56,57,60,61]. In the ZOE-50 and ZOE-70 trials, local reactions such as pain, redness, and swelling were more common in RZV recipients (74–82%) than placebo recipients (10–12%), but most were short-lived and lasted for a median of 2 days [61]. In RCTs of immunocompromised populations, the frequency of local reactions was similar to immunocompetent populations. Systemic reactions such as headache and myalgia were also more frequent in the vaccine than the placebo arm in trials of both immunocompetent and immunocompromised populations (RR 2.28, 95% CI: 1.45–3.65) [60]. Across trials of immunocompetent and immunocompromised populations, no differences in severe adverse events have been observed between RZV and placebo [33,35,64,65,66]. One cohort study on community-dwelling Medicare beneficiaries found an increased risk of Guillain–Barré syndrome in the RZV group compared to ZVL (RR 2.34, 95% CI 1.00–5.41) [67]. However, early monitoring and post-licensure safety surveillance have not confirmed this, nor identified any other SAE signals [61]. Overall, although RZV is a reactogenic vaccine, it is safe, the duration of symptoms is typically short, and it does not translate to reduced uptake of the second dose [61].

Another consideration for policymakers will be the cost effectiveness of the vaccine. The current list price in the US for RZV is $215.51 per dose [68], making it one of the most expensive vaccines available. Despite this, multiple cost-effectiveness modelling studies have shown that its long-term benefits in reducing the incidence and severity of HZ and its complications result in substantial cost savings, directly from reductions in healthcare costs and indirectly from a reduction in productivity losses. A systematic review of cost-effectiveness modelling studies found that 15 of 18 studies showed RZV to be cost effective compared to no vaccination or vaccination with ZVL, and cost-effective when revaccinating cohorts previously vaccinated with ZVL. However, all studies were conducted in North America, Europe, or Asia. Where the vaccine is considered by policymakers, context-specific economic evaluations are critical in order to take informed decisions. The cost-effectiveness will depend on the local epidemiology, healthcare systems, and potential support from non-governmental organizations in reducing the cost of vaccination for lower-income countries. In areas of Africa and South America, where there is a known high burden of disease of other life-threatening vaccine-preventable diseases such as dengue, malaria, and tuberculosis, RZV vaccination is currently unlikely to be a priority for policymakers, particularly given the high cost. RZV has the potential for great public health benefits, but without wider access to affordable vaccines it also has the potential for driving inequality both nationally and internationally.

With this being said, life-course vaccination is recognized as a global health priority, with the Immunization Agenda 2030 (IA2030) calling for the strengthening of immunization policies and service delivery throughout the life-course and for new collaborations and private-sector partnerships to mobilize financing for vaccination of older age groups. HZ vaccination should be considered in the context of an over-all life-course approach of immunization. Ultimately, in developing policy, the results of this review should be considered jointly with local burden of disease data, given that age of disease onset may vary based on temperate or tropical climate, on current VZV policy, and vaccination uptake.

In conclusion, our comprehensive review underscores the significant protection provided by RZV against HZ and its associated complications. It highlights the good safety profile as well as the considerable efficacy and effectiveness of the vaccine, including in individuals with underlying conditions and comorbidities. However, we also demonstrated a lack of evidence from low- and middle-income countries, both on the burden of disease of HZ and on the efficacy and effectiveness of RZV, highlighting the need for improved global surveillance. The global uptake of the vaccine, including in lower resource settings, will depend on additional research as well as on consideration of factors such as the cost-effectiveness, particularly in regions with competing healthcare priorities and limited resources.

## Figures and Tables

**Table 1:** Inclusion and exclusion criteria for rapid review.

	Inclusion	Exclusion
**Population**	General population	
**Intervention**	Two doses of recombinant adjuvanted zoster vaccine	
**Comparison**	No RZV	
**Outcomes**	Herpes zoster, postherpetic neuralgia, herpes zoster ophthalmicus, other HZ-associated complications. Humoral and cellular immune response for follow-up ≥ 5 years and/or co-administration with another vaccine	
**Study design**	RCT or non-randomised	Case reports, case series, reviews, systematic reviews, commentaries, editorials, in vitro only studies, animal studies
**Time frame**	7 August 2012 to 30 September 2023	
**Languages**	English	Other languages

The publication date of the RZV phase 2 trial results was 7 August 2012 [21]. RZV = Recombinant zoster vaccine; RCT = randomized controlled trial.

**Table 2:** PubMed search terms.

Row	Term	Purpose
**1**	“Herpes Zoster” [Mesh] OR zoster[tiab] OR shingle*[tiab]	Mesh and title/abstract terms for herpes zoster
**2**	Shingrix[tiab] OR RZV OR HZ/su[tiab] OR “recombinant vaccine”[tiab:~3] OR “subunit vaccine”[tiab:~3] OR “glycoprotein E vaccine”[tiab:~3] OR “gE vaccine”[tiab:~3] OR “inactivated vaccine”[tiab:~3] OR “adjuvant* vaccine”[tiab:~3]	Title/abstract terms for Shingrix
**3**	(“7 August 2012”[Date—Publication]: “30 September 2023”[Date—Publication]	Limit publications to those published when the vaccine was in late-stage development or implementation, up to the date of the literature search
**4**	(#1 AND #2 AND #3) NOT (case reports[Publication Type] NOT systematic review[Publication Type]	Exclude reviews and case reports

**Table 3:** Characteristics of studies reporting vaccine efficacy of recombinant zoster vaccine against herpes zoster and its complications.

Author(s), Year	Study Acronym or NCT Number ^1^	N Enrolled	Study Design	# Centres/Location	Study Start—Study End Year	Follow-Up	Population	Risk of Bias
**Lal et al., 2015 [23]** **Cunningham et al., 2016 [24]** **Kovac et al., 2018 [25]** **Willer et al., 2019 [26]** **Curran et al., 2021 [27]** **Dagnew et al., 2021 [28]** **Kim et al., 2021 [29]** **Boutry et al., 2022 [30]** **Strezova et al., 2022 [31]** **Oostvogels et al., 2019 [32]**	ZOE-50	15,411	Phase 3 RCT	248/Europe, North America, Latin America, and Asia	Enrollment: 2010–2011	Mean 3.5 years	Immunocompetent adults, 50+ years old	Low
**Cunningham et al., 2016 [24]** **Kovac et al., 2018 [25]** **Willer et al., 2019 [26]** **Curran et al., 2021 [27]** **Dagnew et al., 2021 [28]** **Kim et al., 2021 [29]** **Boutry et al., 2022 [30]** **Strezova et al., 2022 [31]** **Oostvogels et al., 2019 [32]**	ZOE-70	13,900	Phase 3 RCT	248/Europe, North America, Latin America, and Asia	Enrollment: 2010–2011; Last study visit: 2015	Mean 3.7 years	Immunocompetent adults, 70+ years old	Low
**Bastidas et al., 2019 [33]** **Stadtmauer et al., 2021 [34]**	ZOE-HSCT	1868	Phase 3 RCT	167/Multi	2010–2017	Median 1.8 years	HSCT recipients, 18+ years old	Low
**Dagnew et al., 2019 [35]**	Zoster-039	606	Phase 3 RCT	77/Europe, North America, Latin America, and Asia	Enrollment: March 2013–September 2015	Median 11.1 months	Hematological malignancy patients	Some concerns
**Boutry et al., 2022 [30]** **Strezova et al., 2022 [31]**	ZOE-LTFU ^2^	7413	Phase 3B open-label single arm	248/Europe, North America, Latin America, and Asia	2016-Present	Up to 6 years ^3^	Immunocompetent adults, 50+ years old	Low
**Curran et al., 2021 [27]**	ZOE-Frailty ^2^	26,976	Phase 3 RCT	248/Europe, North America, Latin America, and Asia	Enrollment: 2010–2011	Mean 4 years	Frail immunocompetent adults, 50+ years old	Low

NCT = National clinical trial, ZOE = Zoster efficacy study, LTFU = long-term follow-up. ^1^ NCT Number is the ClinicalTrials.gov Identifier: KA-03909·NLM Customer Support Center (nih.gov (accessed on 19 February 2025)). ^2^ ZOE-LTFU (NCT02723773) is a follow-up study and ZOE-Frailty (NCT03563183) is a subgroup analysis of the ZOE-50 (NCT01165177) and ZOE-70 (NCT01165229) studies. ^3^ Beginning of follow-up was 4 years post-vaccination.

**Table 4:** Characteristics of studies reporting vaccine effectiveness of Recombinant Zoster Vaccine against herpes zoster and its complications.

Author(s), Year	N Enrolled	Study Design	Location	Study Start—Study End Year	Follow-Up	Population	Risk of Bias
**Sun et al., 2021 (A) [36]**	78,358	Retrospective cohort	Hawaii	2018–2019	Mean 0.7 years	Immunocompetent adults, 50+ years old	Moderate
**Kochhar et al., 2021 [37]**	112,220	Retrospective cohort	US	2017–2020	Minimum 9 months	Inflammatory bowel disease patients, 50+ years old	Serious
**Izurieta et al., 2021 [38]**	15,589,546	Prospective cohort	US	2017–2019	Median 3.1 years	General population, 65+ years old	Moderate
**Sun et al., 2021 (B) [39]**	4,769,819	Retrospective cohort	US	2018–2019	Median 7 months	Immunocompetent adults, 50+ years old	Moderate
**Lu et al., 2021 [40]**	4,842,579	Retrospective cohort	US	2018–2019	Median 730 days	Immunocompetent adults, 50+ years old	Moderate
**Bruxvoort et al., 2022 [41]**	41,251	Retrospective cohort	US	2019–2020	Minimum 1 year	Immunocompetent adults, 50+ years old	Low
**Khan et al., 2022 [42]**	33,300	Retrospective cohort	US	2018–2020	Mean 1.13 years	Inflammatory bowel disease patients, 50+ years old	Low
**Parameswaran et al., 2023, A [44]**	2,165,505	Retrospective case–control	US	2010–2020	30 days following HZ infection	General population, 18+ years old	Moderate
**Parameswaran et al., 2023, B [43]**	2,165,584	Retrospective cohort	US	2015–2020	30 days following HZ infection	General population, 18+ years old	Moderate

**Table 5:** Characteristics of studies reporting on the immune response to the recombinant zoster vaccine.

Author(s), Year	Study Acronym or NCT Number	N Enrolled	Study Design	# Centres/Location	Study Start—Study End Year	Follow-Up	Population	Co-Administered Vaccine
**Co-administration**								
**Schwarz, 2017 [45]**	NCT01954251	829	Phase 3 Randomized open label trial	20/Canada, Germany, US	2013–2015	14–16 months	Immunocompetent, 50+ years	Quadrivalent seasonal inactivated influenza vaccine (IIV4)
**Maréchal, 2018 [46]**	NCT02045836	865	Phase 3 Randomized open label trial	9/US, Canada, Estonia	2014–2016	12 months	Immunocompetent, 50+ years	23-valent pneumococcal polysaccharide vaccine (PPSV23)
**Strezova, 2019 [47]**	NCT02052596	904	Phase 3 Randomized open label trial	13/US	2014–2016	12 months	Immunocompetent, 50+ years	Diphtheria-tetanus-acellular pertussis vaccine (Tdap)
**Min, 2022 [48]**	NCT03439657	912	Phase 3B Randomized open label trial	13/Canada, Estonia, Germany, US	2018–2020	12 months	Immunocompetent, 50+ years	13-valent pneumococcal conjugate vaccine (PCV13)
**Naficy, 2023 [49]**	NCT05047770	454	Phase 3 Randomized open label trial	47/US	2021–2022	34 weeks	Immunocompetent, 50+ years	Coronavirus Disease 2019 mRNA-1273
**Galgani, 2023 [50]**	NCT03894969	541	Phase 2a Randomized open label trial	14/Estonia, Finland, France, Italy, Spain	2019–2021	Up to 6 months	Healthy adults 50–80 years with history of smoking	Non-typable Hemophilus influenzae–Moraxella catarrhalis (NTHi-Mcat)
**Long-term (≥5 years) follow-up**								
**Chlibek, 2016 [51]** **Schwarz, 2018 [52]** **Hastie, 2021 [53]**	NCT02735915	70	Phase 3B open-label single arm follow-up study	7/Czech Republic, Germany, Sweden	2016–2018 ^2^	108 to 120 months after first vaccine dose	Immunocompetent adults, 60+ years old	NA
**Boutry, 2022 [30]** **Strezova, 2022 [31]**	ZOE-LTFU ^1^	7413	Phase 3B open-label single arm	248/Europe, North America, Latin America, and Asia	2016-Present	Up to 10 years after first vaccine dose	Immunocompetent adults, 50+ years old	NA
**Johnson, 2022 [54]** **Weinberg, 2023 [55]**	NCT02114333	159	Phase 1 RCT	1/US	2014–2020	60 months	Immunocompetent adults, 50–85 years	NA

NCT = national clinical trial, ZOE = zoster efficacy study, LTFU = long-term follow-up. ^1^ ZOE-LTFU is follow-up study of ZOE-50 and ZOE-70 studies. ^2^ Original study conducted 2016–2018. This study started after 9 years of follow-up.

## Data Availability

Data were retrieved from the articles mentioned in the references.

## Supplementary Materials

The following supporting information can be downloaded at: https://www.mdpi.com/article/10.3390/vaccines13030250/s1, Table S1. Critical appraisal results using the Cochrane Collaboration Risk of Bias 2 Assessment tool. Table S2. Critical appraisal results using the ROBINS-I tool (Risk Of Bias In Non-randomized Studies—of Interventions).
