# Supplementary material for: Evaluating the Immunogenicity, Efficacy, and Effectiveness of Recombinant Zoster Vaccine for Global Public Health Policy
Source: Vaccines (Basel). 2025 Feb 27;13(3):250. doi: 10.3390/vaccines13030250 (PMC11946835; doi:10.3390/vaccines13030250)
Supplement: Supplementary file 1 [file vaccines-13-00250-s001.zip › vaccines-3477144-supplementary.pdf]

Supplementary Materials

Supplementary Methods

Assessment of risk of bias in included studies

Eligible randomized controlled trials (RCTs) were critically appraised by one reviewer (MM) using the standardized critical appraisal tool, Cochrane risk of bias assessment tool (Higgins 2016). Outcomes assessed were efficacy against herpes zoster as well as efficacy against post-herpetic neuralgia.

We evaluated the following domains:

1. Risk of bias arising from the randomization process

2. Risk of bias due to deviations from the intended interventions

3. Missing outcome data.

4. Risk of bias in measurement of the outcome

5. Risk of bias in selection of the reported results

We classified each of these domains as well as the overall risk of bias as 'low risk of bias', 'some concerns', or 'high risk of bias'.

Equally, eligible observational studies were critically appraised by two independent reviewers (RA/ MM) using the standardized critical appraisal tool, ROBINS-I tool (“Risk Of Bias In Non-randomized Studies - of Interventions”). We evaluated the following domains as outlined in the tool:

Pre-intervention

- Bias due to confounding
- Bias in selection of participants into the study

At intervention

- Bias in classification of interventions

Post-intervention

- Bias due to deviations from intended interventions
- Bias due to missing data
- Bias in measurement of outcomes
- Bias in selection of the reported result

Supplementary Tables

Supplementary Table S1. Critical appraisal results using the Cochrane Collaboration Risk of Bias 2 Assessment tool.

Study	Risk of bias	Judgment
Lal 2015	Low	Low risk of bias
Cunningham 2016	Low	Low risk of bias
Dagnew 2019	Some concerns	Some concerns due to post-hoc analysis
Willer 2019	Low	Low risk of bias
Kim 2021	Low	Low risk of bias
Boutry 2022	Low	Low risk of bias
Strezova 2022	Low	Low risk of bias
Dagnew 2021		
Oostvogels 2019	Low	Low risk of bias

Supplementary Table S2. Critical appraisal results using the ROBINS-I tool (Risk Of Bias In Non-randomized Studies - of Interventions)

Study	Risk of bias	Judgement
Sun, Jackson (A), 2021	Moderate	Moderate risk of bias (the study appears to provide sound evidence for a non-randomized study but cannot be considered comparable to a well-performed randomized trial)
Izurieta, 2021	Moderate	Moderate risk of bias (the study appears to provide sound evidence for a non-randomized study but cannot be considered comparable to a well-performed randomized trial)
Sun, Kim (B), 2021	Moderate	Moderate risk of bias (the study appears to provide sound evidence for a non-randomized study but cannot be considered comparable to a well-performed randomized trial)
Lu 2021	Moderate	Moderate risk of bias (the study appears to provide sound evidence for a non-randomized study but cannot be considered comparable to a well-performed randomized trial)
Parameswaran 2023	Moderate	Moderate risk of bias (the study appears to provide sound evidence for a non-randomized study but cannot be considered comparable to a well-performed randomized trial)
Khan 2022	Low	Low risk of bias (the study is comparable to a well-performed randomized trial)
Kochhar 2021	Serious	Serious risk of bias (the study has some important problems)
Bruxvoort 2022	Low	Low risk of bias (the study is comparable to a well-performed randomized trial)
